# Comparative Investigation of Cardiac Injury Mediated by IL-40 and Oxidative Stress Markers in Pericardial Fluid and Serum

**DOI:** 10.3390/medicina61081448

**Published:** 2025-08-12

**Authors:** Murat Ziya Bağış, Yasemin Hacanlı, İsmail Koyuncu, Kadir Eği, Bişar Amaç

**Affiliations:** 1 Department of Cardiovascular Surgery, Faculty of Medicine, Harran University, Sanliurfa 63300, Turkey; ziyabagis@hotmail.com; 2 Department of Perfusion, Faculty of Health Sciences, Harran University, Sanliurfa 63300, Turkey; yaseminhacan@hotmail.com; 3 Department of Medical Biochemistry, Faculty of Medicine, Harran University, Sanliurfa 63300, Turkey; ismailkoyuncu1@gmail.com; 4 Department of Medical Biochemistry, Faculty of Medicine, Gaziantep University, Gaziantep 27310, Turkey

**Keywords:** cytokines, cytokines and cardiovascular diseases, interleukin-40, pericardial fluid and cytokines

## Abstract

*Background and Objectives*: The aim of this study was to investigate IL-40 levels in preoperative blood and intraoperative pericardial fluid samples obtained from healthy individuals and patients with diagnosed heart disease who were scheduled for open heart surgery to determine whether it is a biomarker for cardiovascular diseases. *Material and Methods*: A total of 90 individuals were included in the study and divided into three groups. Group 1 (Healthy Group, n = 45): Blood was collected from a total of 45 healthy men and women over 18 years of age without any diagnosis of cardiovascular disease. Group 2 (Patient Group 1, n = 45): In this group, blood samples from a total of 45 male and female patients over 18 years of age with a diagnosis of heart disease and scheduled for open heart surgery were studied. Group 3 (Patient Group 2, n = 45): Pericardial fluids were obtained from patients in Group 3 immediately after pericardial incision during surgery. IL-40, TAS, TOS and OSI levels in blood plasma and pericardial fluid were determined by the ELISA kit method. *Results*: In the statistical analysis between the groups, IL-40, TOS and OSI levels were found to be higher in the patient group and in the pericardial fluid (*p* < 0.001), while TAS was higher in the control group. It was considered statistically significant (*p* < 0.001). *Conclusions*: IL-40, TOS and OSI levels were elevated in patient serum and pericardial fluid. Therefore, we suggest that IL-40 may be a new biomarker for the detection of cardiovascular diseases.

## 1. Introduction

Cardiovascular diseases (CVDs) are disorders of the heart and blood vessels. Early diagnosis and treatment of CVDs can be life-saving. Cytokines, both pro-inflammatory and anti-inflammatory, play diverse roles in the pathophysiology of cardiovascular diseases (CVDs), influencing both disease progression and modulation. They may exert this activity by inhibiting endothelium-dependent vasodilation, altering vasoconstriction and regulating the inflammatory response [1].

Cytokines are glycoproteins or polypeptides weighing 6 to 70 kDa. They are responsible for differentiation, proliferation, function and survival of the target cell [2]. Cytokines can induce or maintain the inflammatory response by reacting at the site of plaque or tumor formation [3]. Many cytokines are known to be associated with the incidence of CVD in a logarithmic pattern [4].

Cytokines are divided into different classes including growth factors, interferons (IFNs), tumor necrosis factor (TNF) family, interleukins (ILs) and chemokines [5]. Recently discovered cytokines include IL-30 and IL-40. IL-40 is not included in any cytokine superfamily. ILs included in the IL-1 superfamily include IL-33, IL-36, IL-37 and IL-38, while new members of the IL-12 family include IL-30, IL-35 and IL-39. Other ILs (IL-31, IL-32, IL-34 and IL-40) are encoded by genes that do not belong to any cytokine superfamily [6]. In previous studies, IL-40 was reported to be produced in diseases related to B cells, but recent studies suggest that it is also synthesized by immune cells other than B cells. This cytokine has been shown to play a role in different functions in the living body. These functions include the generation of B cells in the bone marrow, IgA synthesis and IgA expression in the gut microbiome. It has also been shown to be associated with many autoimmune and inflammatory diseases such as rheumatoid arthritis, diabetes, lymphoma and ankylosing spondylitis [7].

Early diagnosis of CVDs will help treat or prevent these diseases. New biomarkers are therefore needed. Both blood samples and pericardial fluid are used to identify these markers. Pericardial fluid consists of plasma ultrafiltrate originating in the cardiac interstitium and extending from the epicardial layer to the pericardial sac. The pericardial sac, which contains the pericardial fluid, contains epicardial adipose tissue. This tissue is critical for metabolic, hormonal and biochemical regulation of cardiac homeostasis. This importance has led to the recognition of pericardial fluid as a potential marker for the diagnosis and prognosis of different cardiac disorders such as heart failure, cardiac arrhythmias and myocardial ischemia [8].

According to our literature review, the association of IL-40 with CVDs has not been investigated. The aim of this study was to investigate IL-40 levels in preoperative blood and intraoperative pericardial fluid samples obtained from healthy individuals and patients with diagnosed heart disease who were scheduled for open heart surgery and to determine whether it is a biomarker for CVDs.

## 2. Materials and Methods

### 2.1. Ethical Approval

Our study was conducted with the approval of the Harran University Clinical Research Ethics Committee (Date: 16 June 2025, Decision no: HRÜ/25.11.04).

### 2.2. Working Groups

A total of 90 individuals were included in the study. These individuals were divided into three groups as Healthy Group, Patient Group 1 and Patient Group 2.

Group 1 (Healthy Group, n = 45): Blood was collected from a total of 45 healthy men and women over 18 years of age without any diagnosis of cardiovascular disease.

Group 2 (Patient Group 1, n = 45): In this group, blood samples taken for routine procedures of 45 male and female patients over 18 years of age who were diagnosed with heart disease and planned for open heart surgery in the Cardiovascular Surgery Clinic of Harran University Faculty of Medicine Hospital were studied. Blood samples were taken one day before surgery.

Group 3 (Patient Group 2, n = 45): Patients in Group 3 had their pericardial fluids removed immediately after pericardial incision during surgery.

Exclusion criteria included acute infection within the last 4 weeks, known autoimmune or chronic inflammatory diseases, rheumatoid arthritis, osteoarthritis, chronic kidney or liver failure, active malignancy, recent major surgery or trauma (within the last 3 months), use of systemic corticosteroids or immunosuppressive agents, and incomplete clinical data. Current smokers and pregnant women were also excluded from the study.

### 2.3. Obtaining Blood Plasma and Study Methods

Blood samples taken from healthy individuals and blood samples taken for routine procedures from patients with diagnosed heart disease who were scheduled for open heart surgery were transferred to anticoagulated sterile biochemistry tubes. These blood samples from both groups were centrifuged at 4000 rpm for 10 min. After centrifugation, the plasma portion of the blood samples was transferred to Eppendorf tubes and stored at −80 °C until the study day. At the same time, the pericardial fluid obtained during the surgical operation of patients undergoing open heart surgery was transferred to sterile biochemistry tubes without anticoagulant. This fluid was centrifuged at 4000 rpm for 10 min. The supernatant was placed in Eppendorfs. On the study day, Eppendorfs containing all blood and pericardial samples were removed from −80 °C and thawed at room temperature. IL-40 level and oxidative stress markers total antioxidant status (TAS) and total oxidant status (TOS) levels were measured. While these measurements were performed, IL-40 (Cat.No: EH5091) was analyzed by the ELISA kit method. Total antioxidant status (TAS), total oxidant status (TOS) and oxidative stress index (OSI) values [9,10] were measured by the Rel Assay Diagnostic kit method. TAS, TOS and OSI were measured to evaluate the oxidative stress profile of patients with cardiovascular disease, which is a known contributing factor in CVD pathogenesis.

### 2.4. Statistical Analysis

Statistical analyses of the study data were performed using SPSS (Statistical Package for the Social Sciences) version 27.0 (IBM Corp., Armonk, NY, USA) and MetaboAnalyst 6.0. Quantitative data are expressed as mean ± standard deviation (mean ± SD). One-way analysis of variance (one-way ANOVA) was used to compare the three different groups (control, patient serum and pericardial fluid). Tukey’s post hoc test was applied to determine which groups the differences were between for the variables for which significant differences were found as a result of ANOVA. VIP (variable importance in projection) scores were evaluated and PLS-DA (partial least squares discriminant analysis) analysis was performed to determine the main discriminators between variables. In addition, two-dimensional and three-dimensional PCA (principal component analysis) analyses and biplot and heatmap graphs were used to visualize the differences in biochemical profiles between sample groups. The significance level was accepted as *p* < 0.05 in all statistical tests.

## 3. Results

Demographic data of the patients are given in Table 1a,b. According to these data, no significant difference was observed between the groups in terms of age (57.71 ± 10.34), height (1.68 ± 0.09) and weight (80.07 ± 12.70) in Table 1a (*p* > 0.05). Table 1b shows that 15 of 45 patients in the patient group were female (33.33%) and 30 were male (66.67%), while 18 of 45 patients in the control group were female (40%) and 27 of 45 patients were male (60%). In addition, the groups were similar in terms of smoking, with 60 (60%) smokers and 40 (40%) non-smokers.

In Table 2, statistical analysis of different parameters in the control (A), patient serum (B) and pericardial fluid (C) groups was performed. According to this analysis, IL-40, TAS, TOS and OSI values were statistically significant between the groups (*p* < 0.001). IL-40 levels were higher in the patient serum (289.77 ± 129.72 PG/ML) and pericardial fluid (1434.65 ± 342.4 PG/ML) compared to the control group (172.33 ± 65.29 PG/ML). It was considered statistically significant (*p* < 0.001). The TAS level was highest in the control group (2.03 ± 0.63 mmol Trolox Equivalent/L) (*p* < 0.001). TOS was highest in the pericardial fluid (14.87 ± 5.32 μmol H_2_O_2_ Equivalent/L) and patient serum (14.84 ± 2.21 μmol H_2_O_2_ Equivalent/L) and lowest in the control group (13.56 ± 1.23 μmol H_2_O_2_ Equivalent/L). It was considered statistically significant (*p* < 0.001). OSI levels were also found to be significant between the groups in parallel with TAS and TOS values (*p* < 0.001).

**Figure 1:**
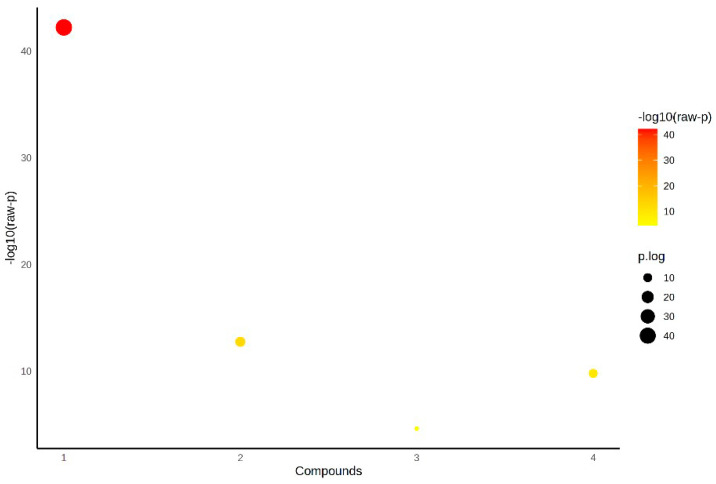
Statistical analysis of parameters between groups by ANOVA.

Figure 1 presents the statistical analysis of IL-40, TOS, TAS and OSI parameters between the control, patient serum and pericardial fluid groups using ANOVA. The color and size of the bubbles represent the level of statistical difference among these parameters (Figure 1).

**Figure 2:**
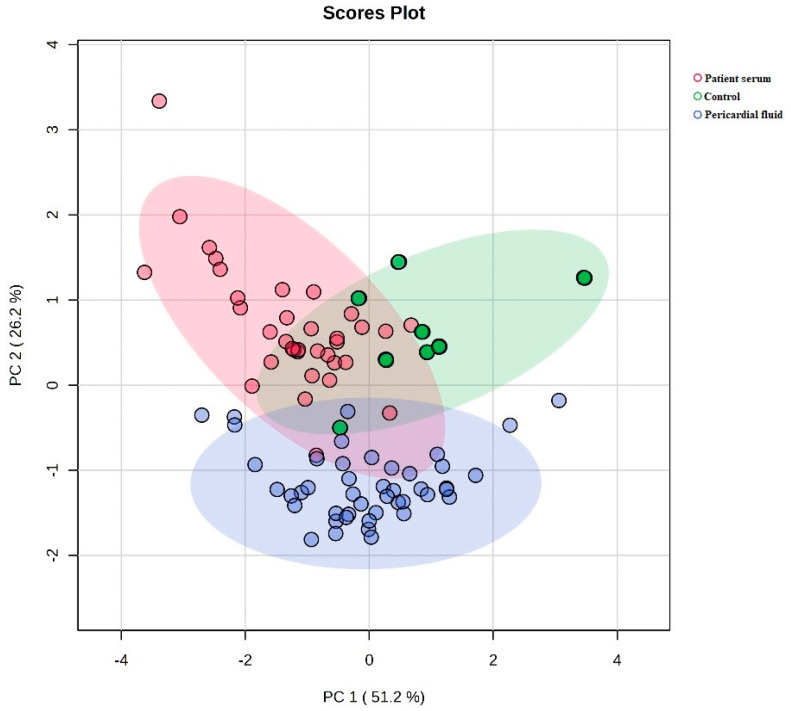
Comparison of metabolic profiles between groups by two-dimensional PCA plot.

Figure 2 shows a two-dimensional principal component analysis (PCA) plot comparing the metabolic profiles of the control, patient serum and pericardial fluid groups. The first component (PC1) accounts for 51.2% of the total variance, and the second component (PC2) accounts for 26.2%, explaining 77.4% of the total variance together. The control group is separated from the patient serum and pericardial fluid groups, while the patient serum and pericardial fluid groups are positioned closer to each other on the PCA plane.

**Figure 3:**
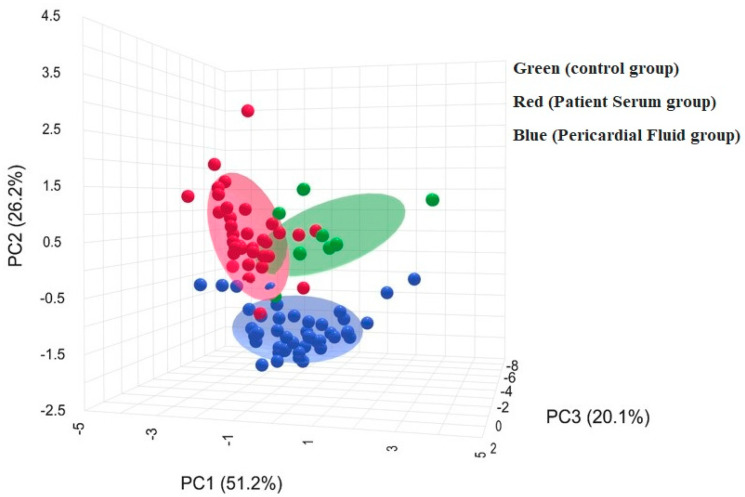
Biochemical profiles of groups by three-dimensional PCA plot.

Figure 3 illustrates a three-dimensional PCA plot with PC1, PC2 and PC3 explaining 51.2%, 26.2% and 20.1% of the total variance, respectively. The control group is located in a distinct cluster, whereas patient serum and pericardial fluid groups are positioned in a different region, with partial overlap between them.

**Figure 4:**
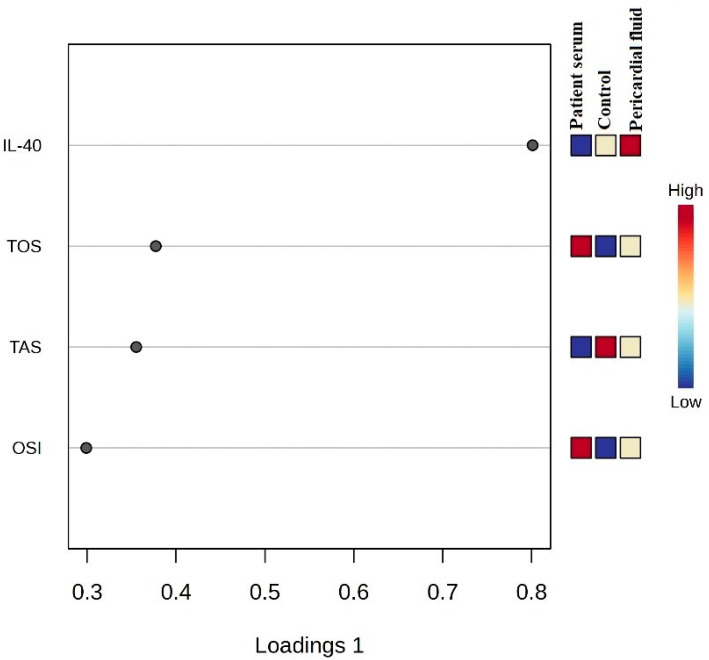
VIP score plot and biochemical differentiation between groups.

Figure 4 displays the variable importance in projection (VIP) scores obtained from the PLS-DA analysis. IL-40, TOS, TAS and OSI have the highest VIP scores, indicating their contribution to the group separation.

**Figure 5:**
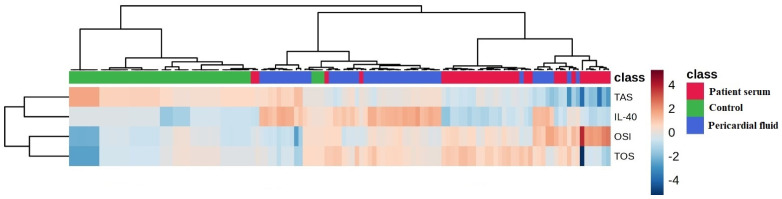
Comparison of IL-40, TAS and TOS levels in groups by heatmap graphic.

Figure 5 presents a heatmap comparing the levels of IL-40, TAS and TOS across the three groups. Color intensities correspond to the measured concentration levels.

**Figure 6:**
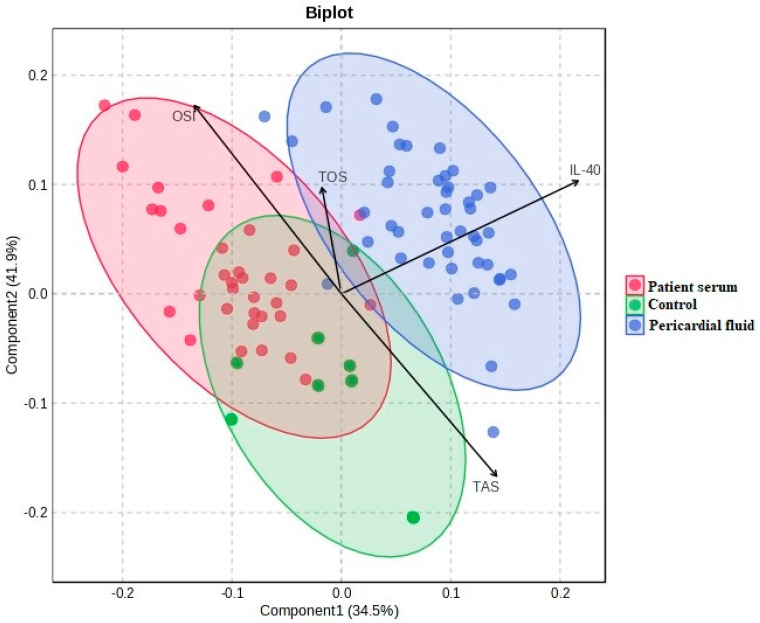
PCA biplot and differentiation of groups on IL-40, TOS, TAS and OSI parameters.

Figure 6 shows the PCA biplot where the IL-40, TOS, TAS and OSI variables are used to illustrate group distribution. The pericardial fluid group is located in a distinct region, while the patient serum and control groups show partial overlap. The variable vectors indicate the direction and magnitude of each parameter’s contribution to the model.

## 4. Discussion

Cytokines, both pro-inflammatory and anti-inflammatory, play diverse roles in the pathophysiology of CVDs, influencing both disease progression and modulation [1]. Recently discovered cytokines include IL-30 and IL-40 [6]. IL-40 has been shown to be associated with diseases such as rheumatoid arthritis, diabetes, lymphoma and ankylosing spondylitis [7]. Navrátilová et al., in their study investigating the function of IL-40 on rheumatoid arthritis, examined IL-40 levels in synovial fluid in healthy, osteoarthritic, and rheumatoid arthritis groups. It was explained that IL-40 played a role in the upregulation of matrix metalloproteinase (MMP)-13 and chemokine in synovial fibroblasts and also had a function in the regulation of tissue destruction and inflammation in rheumatoid arthritis [11]. Navrátilová et al., since IL-40 has a function in the development of rheumatoid arthritis in neutrophils, examined IL-40 in the early stages of rheumatoid arthritis. IL-40 was analyzed in sera of patients with untreated early rheumatoid arthritis, 3 months after the start of treatment and in healthy controls. They reported that IL-40 was elevated in seropositive patients with early rheumatoid arthritis and decreased after treatment. Therefore, they explained that IL-40 may have a function in early rheumatoid arthritis [12].

In a study comparing IL-40 levels in healthy individuals and patients with rheumatoid arthritis, serum IL-40 levels were found to be higher in patients with rheumatoid arthritis compared to healthy individuals. Correlation analyses revealed that serum IL-40 levels were positively correlated with auto-antibodies and coagulation markers associated with rheumatoid arthritis. It has also been reported that serum IL-40 levels are lower in patients with rheumatoid arthritis, comorbidities with osteoarthritis, or comorbidities with cardiovascular diseases. However, it was explained that the underlying mechanism was not fully understood [13]. Jaber et al. investigated the levels of some cytokines including IL-40 in patients with ankylosing spondylitis. According to the results of this study, it was observed that the IL-40 level was upregulated in patients with ankylosing spondylitis independently of factors such as disease duration, age at onset of the disease, patient age, disease activity, etc. [14]. In the study investigating whether IL-40 is a biomarker in type II diabetes, an enzyme-linked immunosorbent assay was used. According to the data of the study, it was observed that there was a significant increase in the IL-40 level in the sera of the patient group compared to the control group [15]. This study conducted in 2023 is the only study that has investigated the relationship between type II diabetes and IL-40. An IL-40 increase in the patient group was reported to be a 53.36-fold increased risk compared to the control group [7]. In a study examining the expression of IL-40 in primary Sjögren’s syndrome and Sjögren’s syndrome-related lymphomas, 29 patients and 24 controls were included in the study. Salivary gland biopsies were obtained from both groups and parotid gland samples were obtained from Sjögren’s syndrome-related lymphomas. As a result of the investigations, it was explained that IL-40 may have a function in the pathogenesis of primary Sjögren’s syndrome and in lymphomas associated with primary Sjögren’s syndrome [16]. Cai et al. investigated whether IL-40 deficiency could provide therapeutic targets in sepsis using WT (IL-40-treated mice) and IL-40 -/- mice. According to the results of the study, the survival rate of septic mice in the group in which IL-40 was genetically knocked out increased from 20% to 60%. In other words, it decreased the mortality rate [17]. In a study investigating the possibility of IL-40 as a marker in systemic lupus erythematosus, an autoimmune disease, IL-40 levels were higher in patients with systemic lupus erythematosus than in controls. Therefore, IL-40 was thought to be associated with both systemic lupus erythematosus and increased disease severity [18]. Bagriacik et al. compared serum IL-40 levels in healthy individuals with serum IL-40 levels in patients with both COVID-19 and pneumonia symptoms. Their study was the first to show that SARS-CoV-2 infection significantly increases serum IL-40 levels. It was explained that the advanced stage of the infection caused an increase in serum IL-40 levels. Therefore, IL-40 levels were also measured to be high in patients with pneumonia symptoms [19]. 

Pericardial fluid is considered to be a potential marker for the diagnosis and prognosis of different cardiac disorders such as heart failure, cardiac arrhythmia and myocardial ischemia [8]. Some inflammatory cytokines such as IL-1, IL-6, IL-8, IL-10, IFNγ and TNF-α have been examined in pericardial fluid [20]. However, IL-40 level in pericardial fluid was not investigated in our literature review.

Oxidative stress is considered an important mechanism in the context of cardiovascular conditions, particularly due to its involvement in tissue damage and altered cellular homeostasis. In this study, oxidative status was evaluated using TAS, TOS and OSI parameters. TAS reflects the plasma’s total capacity to counteract free radicals and oxidative molecules, whereas TOS indicates the cumulative amount of oxidative compounds present in the biological sample. OSI, calculated as the TOS/TAS ratio, serves as an integrated indicator of the balance between oxidant load and antioxidant defense mechanisms. The data obtained from our analysis revealed a clear decrease in TAS levels in both patient serum and pericardial fluid samples compared to healthy controls, pointing toward a reduction in antioxidant potential in individuals with cardiovascular disease. In parallel, TOS and OSI levels were markedly elevated in the patient groups, particularly in pericardial fluid, indicating an increased oxidative burden. This observation suggests that the local cardiac environment, as reflected by pericardial fluid composition, may exhibit higher oxidative stress than the systemic circulation. The consistent pattern of decreased TAS and increased TOS/OSI in patient samples supports the presence of an oxidative imbalance, which may contribute to the pathophysiological alterations observed in cardiovascular conditions. Furthermore, the marked oxidative changes detected in pericardial fluid may reflect local inflammatory and degenerative processes at the tissue level. In this context, TAS, TOS and OSI measurements can provide valuable insight into the oxidative profile of cardiac patients and may potentially assist in monitoring disease-related biochemical alterations.

It should be noted that changes in IL-40 and TAS levels may not be exclusively linked to cardiovascular pathology. These markers can also be influenced by broader systemic conditions such as aging, smoking status or low-grade chronic inflammation.

### Limitations

This study has certain limitations. The relatively limited sample size may restrict the generalizability of the results. Future research with larger cohorts and a longitudinal design would be valuable to confirm our findings and to better understand the dynamic behavior of the biomarker over time.

## 5. Conclusions

Early diagnosis of CVDs will help treat or prevent these diseases. Therefore, new biomarkers are needed. We performed this study in both patient sera and pericardial fluid to determine whether IL-40 is a biomarker for CVDs, because according to our literature review, the relationship of IL-40 with CVDs has not been investigated. In our study, IL-40, TOS and OSI levels were measured to be high in patient serum and pericardial fluid. In light of these data, we suggest that IL-40 may be a new biomarker for the detection of CVDs.

## Figures and Tables

**Table 1:** (**a**). Demographic data. (**b**). Patients’ gender and smoking status.

**(a)**							
	**Patient**			**Control**			
	**Min**	**Max**	**Ort ± SS**	**Min**	**Max**	**Ort ± SS**	***p***
Age	27	77	57.71 ± 10.34	28	52	40.31± 9.69	0.06
Height	1.5	1.85	1.68 ± 0.09	1.56	1.85	1.71 ± 0.11	0.052
Weight	57	114	80.07 ± 12.70	66	88	78.78 ± 8.20	0.32
**(b)**							
				**Patient (%)**	**Number**	**Control (%)**	**Number**
Gender		Female		33.33	15	40	18
		Male		66.67	30	60	27
Smoking		Yes		60	27	60	27
		No		40	18	40	18
		Total		100	45	100	45

**Table 2:** Statistical analysis between groups.

	Control (A)			Patient Serum (B)			Pericardial Fluid (C)				
	Min	Max	Ort ± SS	Min	Max	Ort ± SS	Min	Max	Ort ± SS	*p* ^a^	Post Hoc ^b^
IL-40	117.38	613.63	172.33 ± 65.29	124.88	724.88	289.77 ± 129.72	641.13	2039.88	1434.65 ± 342.4	0.001 **	A-B, B-C, A-C
TAS	1.23	3.67	2.03 ± 0.63	0.27	1.75	1.15 ± 0.38	0.73	3.73	1.5 ± 0.49	0.001 **	A-B, B-C, A-C
TOS	11.20	16.32	13.56 ± 1.23	11.20	23.10	14.84 ± 2.21	11.15	44.60	14.87 ± 5.32	0.001 **	A-B, B-C, A-C
OSI	0.42	1.10	0.78 ± 0.17	0.74	4.10	1.46 ± 0.66	0.55	3.65	1.08 ± 0.53	0.001 **	A-B, B-C, A-C

^a^ ANOVA test; *p* < 0.001; ** *p* < 0.05; ^b^ Tukey’s test.

## Data Availability

The data presented in this study are available in this article.
